# Stimulation of noradrenergic transmission by reboxetine is beneficial for a mouse model of progressive parkinsonism

**DOI:** 10.1038/s41598-019-41756-3

**Published:** 2019-03-27

**Authors:** Grzegorz Kreiner, Katarzyna Rafa-Zabłocka, Justyna Barut, Piotr Chmielarz, Marta Kot, Monika Bagińska, Rosanna Parlato, Władysława Anna Daniel, Irena Nalepa

**Affiliations:** 1 Department Brain Biochemistry, Institute of Pharmacology, Polish Academy of Sciences, 31-343 Kraków, Smętna 12, Poland; 2 Department Pharmacokinetics and Drug Metabolism, Institute of Pharmacology, Polish Academy of Sciences, 31-343 Kraków, Smętna 12, Poland; 3 Institute of Applied Physiology, University of Ulm, 89081 Ulm, Germany; 4 Institute of Anatomy and Cell Biology, University of Heidelberg, 69120 Heidelberg, Germany

## Abstract

Parkinson’s disease (PD) is the second most common neurodegenerative disorder and is characterized by motor deficits such as tremor, rigidity and bradykinesia. These symptoms are directly caused by the loss of dopaminergic neurons. However, a wealth of clinical evidence indicates that the dopaminergic system is not the only system affected in PD. Postmortem studies of brains from PD patients have revealed the degeneration of noradrenergic neurons in the locus coeruleus (LC) to the same or even greater extent than that observed in the dopaminergic neurons of substantia nigra (SN) and ventral tegmental area (VTA). Moreover, studies performed on rodent models suggest that enhancement of noradrenergic transmission may attenuate the PD-like phenotype induced by MPTP administration, a neurotoxin-based PD model. The aim of this study was to investigate whether chronic treatment with either of two compounds targeting the noradrenergic system (reboxetine or atipamezole) possess the ability to reduce the progression of a PD-like phenotype in a novel mouse model of progressive dopaminergic neurodegeneration induced by the genetic inhibition of rRNA synthesis in dopaminergic neurons, mimicking a PD-like phenotype. The results showed that reboxetine improved the parkinsonian phenotype associated with delayed progression of SN/VTA dopaminergic neurodegeneration and higher dopamine content in the striatum. Moreover, the alpha1-adrenergic agonist phenylephrine enhanced survival of TH+ neurons in primary cell cultures, supporting the putative neuroprotective effects of noradrenergic stimulation. Our results provide new insights regarding the possible influence of the noradrenergic system on dopaminergic neuron survival and strongly support the hypothesis regarding the neuroprotective role of noradrenaline.

## Introduction

Parkinson’s disease (PD) is the second most common neurodegenerative disorder, affecting up to 3% of elderly populations (>65 years). PD is characterized by motor deficits such as tremor, rigidity and bradykinesia^1^. These symptoms are mostly caused by the loss of dopaminergic neurons located in the substantia nigra (SN) and ventral tegmental area (VTA). Since more than 90% of PD cases are of sporadic origin, few advancements in disease treatment have been made. Over the last 40 years, researchers have been focused on pharmacological enhancement of dopaminergic transmission, which is usually implemented when most of the neurons are already gone.

Nevertheless, PD is associated also with non-dopaminergic neuronal transmission, particularly the extranigral noradrenergic system^2^. Specifically, clinical pathologic examination of human brains has revealed that in PD neuronal loss in the locus coeruleus (LC) is even greater than that in the SN/VTA^3^. Experimental data from animal models also indicate the involvement of noradrenaline (NA) in PD-related brain damage; the loss of NA in PD models can worsen the dopaminergic nigrostriatal damage and conversely, enhanced activity of NA is believed to have a neuroprotective role^4,5^. In particular, after chemical sympathectomy, mice have been reported to be more vulnerable to MPTP (1-methyl-4-phenyl-1,2,3,6-tetrahydropyridine, a neurotoxin used widely for pharmacological modeling of PD)^5^. Moreover, transgenic mice lacking noradrenergic transporters (NET KO mice characterized by enhanced noradrenergic transmission) are more resistant to this compound^4^. NA influences synaptic transmission and plasticity through adrenergic receptors which affects the general behavior. Namely, activation of the alpha_1_-adrenergic receptor (alpha_1_-AR) – resulting in enhancement of noradrenergic transmission – increases locomotor activity, and activation of the alpha_2_-adrenergic receptor (alpha_2_-AR) gives the opposite effects^6^. Moreover, the activation of (alpha_1_-AR) may facilitate dopaminergic transmission in the striatum and ventral midbrain^7^. On the other hand, extracellular NA release is limited through the stimulation of auto-inhibitory alpha_2_-AR, explaining the beneficial role of alpha_2_-AR antagonists on motor function in context of PD symptoms^8^. Overall, these data suggest that impairment of noradrenergic signaling may substantially contribute to various motor and non-motor dysfunction in PD, and that enhancing the function of the noradrenergic system might be a potential promising therapeutic target for treating PD^8–10^. However, the main caveat of these studies focusing on the beneficial role of the noradrenergic signaling in PD was the use of models that did not show progressive neurodegeneration.

This study aimed at understanding whether pharmacological enhancement of noradrenergic transmission achieved either by chronic application of a selective NA reuptake inhibitor (reboxetine, REB) or an alpha_2_-AR antagonist (atipamezole, APM) can ameliorate the motor effects of progressive loss of dopaminergic neurons and whether this noradrenergic stimulation can have any beneficial effects on dopaminergic neuronal survival and striatal dopamine content in a mouse model of progressive parkinsonism.

To test this hypothesis, we used a conditional knock-out mouse model lacking the transcription initiation factor-IA (TIF-IA) characterized by the inducible inhibition of a fundamental cellular function, such as rRNA synthesis in dopaminergic neurons to cause their progressive neurodegeneration^11^. These mutant mice mimic many hallmarks of PD, including progressive and selective vulnerability of SN neurons, motor coordination deficits, as well as increased mitochondrial dysfunction and increased oxidative stress damage^11^. Importantly, as previously shown, some of the effects of this mutation can be partially rescued by L-DOPA administration^11^ as well as by inhibition of pro-apoptotic signaling pathways such as p53 and enhancement of the master regulator of protein synthesis, the mechanistic target of rapamycin (mTOR)^11,12^, supporting their potential use as a model for disease-modifying therapies against dopaminergic neurodegeneration.

## Materials and Methods

### Animals

Selective ablation of TIF-IA in dopaminergic neurons (TIF-IA^DATCreERT2^ mice) was achieved using the *Cre/loxP* approach. Transgenic mice hosting *Cre* recombinase under the dopamine transporter (DAT) promoter (DAT^CreERT2^ mice) were crossed with animals harboring the floxed TIF-IA gene, as previously described^11^. Male and female mutant mice were kept with their control (Cre-negative) littermates of the same sex in self-ventilated cages under standard laboratory conditions (12 h light/dark cycle, food and water *ad libitum*). The study was carried out in strict accordance with the recommendations in the Guide for the Care and Use of Laboratory Animals of the National Institutes of Health. The protocol for all the behavioral studies was approved by the Animal Ethical Committee at the Institute of Pharmacology, Polish Academy of Sciences (Permit Number: 951/2012, issued: 28.06.2012).

### Drugs and experimental schedule

**Figure 1:**
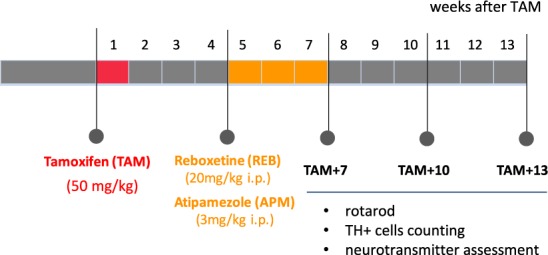
Flowchart summarizing experimental design. TAM – tamoxifen, REB – reboxetine, APM – atipamezole; TAM + 7, TAM + 10, TAM + 13:7, 10, 13 weeks after induction of the mutation (application of tamoxifen).

To activate tamoxifen-dependent CreERT2 recombinase in adult mice (12 weeks old) and induce the mutation, we applied tamoxifen (TAM) (Sigma-Aldrich, USA) dissolved in oil at a dose of 1 mg/mouse, 2× daily for 5 consecutive days, 4 weeks before administration of investigated drugs. Control (w/t) animals received oil alone. Reboxetine (REB) (Tocris Bioscience, USA) and atipamezole (APM) (Antisedan, Orion Pharma, Poland) were administered once daily for 21 consecutive days at doses of 20 mg/kg and 3 mg/kg, respectively. The control groups received 0.9% NaCl. All behavioral and biochemical analyses were performed 7, 10 and 13 weeks after TAM injections (TAM + 7, TAM + 10, TAM + 13), as seen in Fig. 1.

### Rotarod Test

The rotarod test was performed for motor coordination assessment. The test was performed on an accelerated rotarod (Ugo Basile, Italy). The assessment was preceded by a training session one day before the first experiment (5 min on the rotating rod at a constant speed). During the experimental sessions performed on weeks 7, 10 and 13 after tamoxifen injection, the time spent on the accelerating rod (4 to 40 r.p.m. within 5 min) was measured.

### HPLC analysis

The tissue concentrations of noradrenaline (NA), dopamine (DA), 3,4-dihydroxyphenylacetic acid (DOPAC) and homovanillic acid (HVA) were measured using high-performance liquid chromatography with electrochemical detection, as previously described^13^. Briefly, tissue samples of selected brain structures were homogenized in 20 volumes (v/w) of ice-cold 0.1 M HClO_4_. The homogenates were centrifuged at 15,000 × g for 15 min at 4 °C. After centrifugation, the supernatant was filtered through a 0.2 μm membrane (Alltech). The obtained aliquots (5 μl) were injected into the HPLC system, which included an amperometric detector (LC-4C) with a cross-flow detector cell (BAS, IN, USA), a 626 Alltech pump and a GOLD Hypersil analytical column (3 μm, 100 × 3 mm, Thermo Scientific, USA). Column temperature was maintained at 30 °C. An external standard containing NA, DA, and DOPAC at concentrations of 50 ng/ml and HVA at a concentration of 100 ng/ml was used. The mobile phase consisted of 0.1 M KH_2_PO_4_, 0.5 mM Na_2_EDTA, 80 mg/l sodium 1-octanesulfonate and methanol (4%), adjusted to pH 3.7 with 85% H_3_PO_4_. The mobile phase flow-rate was 0.6 ml/min. An electrode potential (3-mm glassy carbon electrode) was set at 0.7 V with a sensitivity of 5 nA/V. Chromax 2007 software (Pol-Lab, Warszawa, Poland) was used for data collection and analysis.

### Immunohistochemistry

The procedure was performed as previously described^14^, with some modifications. Briefly, the brains were removed, fixed for 48 h in 4% paraformaldehyde (PFA), rinsed and transferred to 0.4% PFA. After fixation, the brains were cut on a vibratome (Leica, Germany) into 30-μm sections. Sections from the midbrain including the SN/VTA were incubated overnight at 4 °C with primary anti-tyrosine hydroxylase (TH) (1:500, Millipore, USA, cat no AB1542) antibody. Visualization of antigen-bound primary antibody was carried out using a proper biotinylated secondary antibody followed by incubation with the Avidin/Biotin Complex (ABC; Vector Laboratories, USA) and diaminobenzidine staining (DAB; Sigma, USA). Stained sections were acquired and analyzed under a light microscope (Nikon Eclipse50i, Japan) equipped with a camera and NIS Elements software. Quantification of TH-positive cells (TH+) was performed manually by counting all TH+ cells on adjacent sections from 6 animals of each genotype/treatment in a single-blind experiment.

### Primary embryonic midbrain cell cultures and drug treatment

Primary cultures and their survival were established and investigated as described previously^15^. E13.5 embryos of C57Bl/6J wild type mice (6 months old) were dissected according to Planket *et al*.^16^. 30 000 live cells were plated on 96-well plate pre-coated with poly-L-ornithine in culture medium (Neurobasal Medium + B27 supplement 1x + 2 mM L-Glutamine) and placed in the incubator. After 45 minutes required for cells attached, 100 µl of medium containing vehicle or investigated drugs were added. Drugs being the subject of investigation: GDNF growth factor – a positive control (100 ng/µl; PeproTech), reboxetine (10 µM; Tocris, #1982) and phenylephrine (100 µM; Sigma, P6126). The optimal dosage of investigated pharmaceuticals was chosen upon own experience and available literature^17^. 48 hours after plating half of the medium was exchanged with fresh one containing the respective drug concentrations. 5 days after plating cells were fixed with 4% PFA, immunostained with anti-TH antibody (Millipore, AB1542, 1:2000) and counterstained with DAPI. The plates where imaged by an inverted fluorescent microscope (AxioObserver, Carl Zeiss, Germany) at 5x magnification and the number of surviving TH+ cells in each separate well were counted automatically in an unbiased way with use of the ImageJ software^18^.

### Statistical analysis

Statistical analysis was performed with Graph Pad Prism 7 software. Data were evaluated by 2-way analysis of variance (2-way ANOVA, genotype × treatment) with multiple comparisons of biologically relevant groups (taking into consideration 3 different time points of analysis) followed by Fisher’s LSD post hoc test. P-values lower than 0.05 were considered statistically significant.

## Results

### Treatment with reboxetine ameliorated motor impairment in the TIF-IA^DATCreERT2^ mice

**Figure 2:**
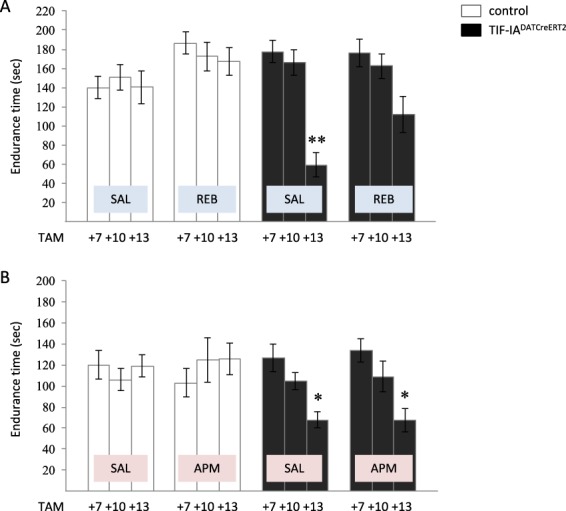
Rotarod test performed on (**A**) reboxetine and (**B**) atipamezole treated TIF-IA^DATCreERT2^ mice. Bars represent time spent on rotating rod until the animal fall down. Data are presented as the mean ± SEM, TAM – tamoxifen, TAM + 7, TAM + 10, TAM + 13-7, 10, 13 days after induction of the mutation (tamoxifen application). Data are the mean ± SEM; **p < 0.01, *p < 0.05 vs control (w/t) group relevant to particular time point; n = (**A**) 24, 18, 10 (TAM + 7, + 10, + 13; respectively); (**B**) 8–10 (all groups).

As indicated in Fig. 1, we induced the conditional ablation of TIF-IA by TAM injection and started the 21-day treatment with either REB or APM 4 weeks after TAM, the time required for effective TAM-induced recombination. To investigate whether REB or APM may have any positive effect on motor deficits triggered by the progressive loss of dopaminergic neurons in TIF-IA^DATCreERT2 mice^, we tested the control and mutant animals at three different time points (7, 10 and 13 weeks after TAM (TAM + 7, TAM + 10 and TAM + 13) by rotarod. Based on previous experiments these were the stages previously shown to lead to a progressive parkinsonism, starting at TAM + 7 (without any cell loss) and developing in severe behavioral phenotype associated with profound dopaminergic degeneration at TAM + 13^11^. As expected, the VEH-treated TIF-IA^DATCreERT2^ mice revealed a significant decrease of endurance (by 57.7% vs control, VEH-treated animals, two-way ANOVA: genotype F_(2,218)_ = 12.63; p < 0.001; *post-hoc* p < 0.01 vs. control group)) on the rotarod, 13 weeks after TAM injection. Interestingly, this reduced endurance did not reach significance when the mutants were exposed to REB (only 19.9% decrease), suggesting that mice treated with REB might display better motor coordination in this test (Fig. 2A). Similar differentiation in attenuation of PD-like symptoms was not observed after APM treatment; the mice exposed to this drug did not differ in the progression of motor deterioration when compared to VEH-treated control animals as measured by the rotarod test. Both groups showed similar decrease of time spent on rotating wheel: 51.7 and 52.1%, respectively (two-way ANOVA: genotype F_(2,89)_ = 3.42; p < 0.05; *post-hoc* p < 0.05) (Fig. 2B).

### Treatment with reboxetine increased the survival of DA neurons in the TIF-IA^DATCreERT2^ mice

**Figure 3:**
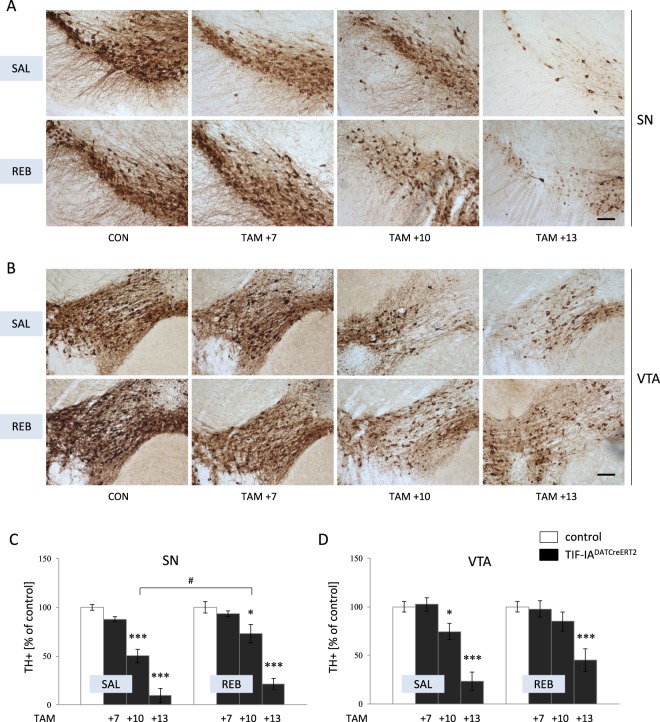
Assessment of dopaminergic neurodegeneration as revealed by TH+ cell immunostaining. Immunohistochemical staining of TH+ cells visualized in (**A**) SN and (**B**) VTA neurons; quantification of TH+ cells performed in (**C**) SN and (**D**) VTA dopaminergic neurons. Bars represent percent of control; TAM – tamoxifen, SAL – saline, REB – reboxetine; TAM + 7, TAM + 10, TAM + 13:7, 10, 13 days after induction of the mutation (tamoxifen application). Data are the mean ± SEM; ***p < 0.001, *p < 0.05 vs control (w/t) group; ^#^p < 0.05 vs TIF-IA^DATCreERT2^ at relevant time point; n = 4.

To further investigate the suggested beneficial impact of REB treatment on dopaminergic neurons survival, we performed immunostaining to identify TH+ cells in the region of SN and VTA (Fig. 3), which confirmed the initial behavioral observations. In particular, TIF-IA^DATCreERT2^ mice treated with REB showed less cell loss at week 10 after tamoxifen induction (TAM + 10). VEH-treated mutant mice were characterized by nearly 50% loss of the TH+ cell compared to control animals, which was significantly lower than that in REB treated mutants (49.7% vs 26.9%; two-way ANOVA: genotype F_(3,24)_ = 84.43; p < 0.001; *post-hoc* p < 0.05) (Fig. 3A,C). At week 13 after mutation induction (TAM + 13), both REB-treated and VEH-treated TIF-IA^DATCreERT2^ mice showed significant loss of TH+ cells, however cell loss was slightly more profound in VEH-treated animals. A similar pattern of degeneration kinetics was observed in the VTA of REB-treated vs. VEH-treated TIF-IA^DATCreERT2^ mice (Fig. 3B,D).

### The effects of reboxetine treatment on dopamine and noradrenaline content in the striatum of TIF-IA^DATCreERT2^ mice

**Figure 4:**
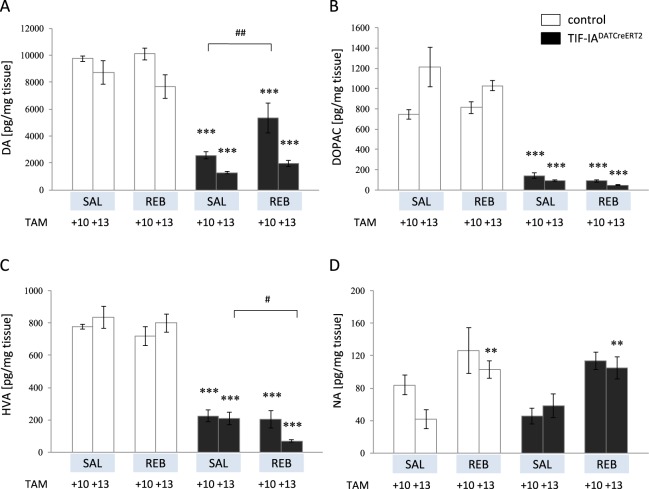
Changes in tissue levels of (**A**) dopamine (DA), (**B,C**) its metabolites (DOPAC, HVA), and (**D**) noradrenaline (NA) levels. Bars represent actual value of each neurotransmitter or its metabolite in pg/mg tissue; TAM – tamoxifen, SAL – saline, REB – reboxetine; TAM + 10, TAM + 13:-10, 13 days after induction of the mutation (tamoxifen application). Data are the mean ± SEM; ***p < 0.001, **p < 0.01 vs appropriate control (w/t) group of relevant time point; ^##^p < 0.01, ^#^p < 0.05 vs TIF-IA^DATCreERT2^ at relevant time point; n = 6.

Since there was no difference between the experimental groups at 7 weeks after TAM (TAM + 7) induction regarding the motor performance and TH+ cells counting parameters, due to limited availability of mutant mice, next we focused on time points TAM + 10 and TAM + 13 to further investigate the beneficial effects of REB on dopamine (DA) level in the striatum of TIF-IA^DATCreERT2^ mice. As expected, the mutation produced a substantial depletion of DA in all animals 13 weeks after tamoxifen induction (TAM + 13) (two-way ANOVA: genotype F_(1,39)_ = 21.86; p < 0.001; *post-hoc* p < 0.001) (Fig. 4A). However, TIF-IA^DATCreERT2^ mice at 10 weeks after tamoxifen induction (TAM + 10) exhibited significantly different levels of DA content, depending on drug treatment (two-way ANOVA: time point F_(3,39)_ = 72.8; p < 0.001; *post-hoc* p < 0.01), which was also somewhat reflected on the levels of the DA metabolite HVA but not DOPAC (Fig. 4A–C). In general, animals receiving REB were also characterized by elevated levels of NA (two-way ANOVA: treatment F_(3,40)_ = 9.08; p < 0.001; *post-hoc* p = 0.051 vs con TAM + 10, p < 0.01 vs con TAM + 13) (Fig. 4D).

### Alpha1-adrenergic agonist phenylephrine revealed pro-survival potential in primary embryonic midbrain cell culture

**Figure 5:**
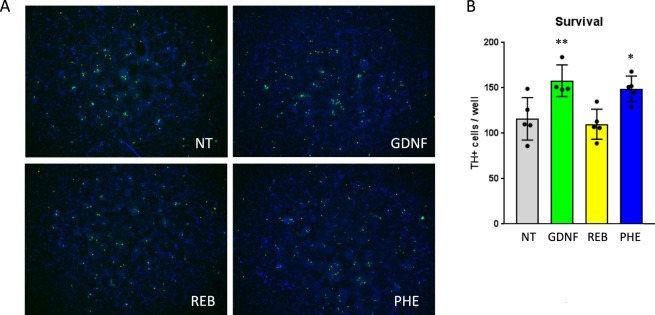
TH+ cells survival in primary embryonic cell culture after exposure to GDNF, reboxetine and phenylephrine. Effects of GDNF administration (100 ng/ml), selective alpha1-AR receptor agonist phenylephrine (100 μM), and reboxetine (10 μM) on TH+ cells survival visualized in representative immunofluorescent labeling (**A**). Quantitative unbiased analysis with ImageJ software (**B**) of the number of TH+ cells per well in each condition. untreated, GDNF, REB and PHE. Data are the mean ± SEM; **p < 0.01, *p < 0.05 vs control group (non-treated medium); n = 4–5 independent wells. NT – non-treated medium, GDNF – glial cell line-derived neurotrophic factor, PHE – phenylephrine, REB – reboxetine.

To test the hypothesis whether the observed beneficial effects of noradrenergic stimulation may have *per se* a neuroprotective potential possibly mediated by alpha1-AR, we performed an additional *in vitro* experiment on primary culture of embryonic midbrain neurons derived from w/t C57Bl/6J mice. Cells were treated with phenylephrine, a selective alpha1-AR receptor agonist. After adding the drug and without further changing the medium, the cells started to die over the time. Phenylephrine increased the survival of TH+ cells in comparison to non-treated cell cultures (Fig. 5A,B). This effect was similar to GDNF growth factor application, serving as a positive control. In particular, phenylephrine direct application to culture medium significantly increased the survival of TH+ cells up to 49% vs non-treated cells, while GDNF treatment effectiveness reached 58% (Fig. 5B). REB treatment did not show any effects on the number of TH+ cells, however it is important to note that REB acts mainly as noradrenergic reuptake inhibitor, with only low affinity to alpha1-AR receptors.

## Discussion

As our previous results showed that some chronically administered antidepressants, which act by augmentation of noradrenergic transmission, enhance expression of alpha_1_-AR^19^ and in light of data suggesting their neuroprotective activity^20^, here, we decided to determine whether chronic treatment with reboxetine, a highly specific noradrenergic reuptake inhibitor, has any positive effect on the time course of symptom progression in a novel model of progressive parkinsonism. Additionally, we extended this investigation by including another drug, atipamezole, an alpha_2_-AR antagonist with potential to enhance noradrenergic transmission but with a different pharmacological mechanism of action by blocking alpha_2_-AR autoreceptors. Experimental animal studies suggest that it might also have beneficial effects in recovery from brain damage and potentiate the antiparkinsonian effects of dopaminergic drugs^21^.

For the purpose of this study, we utilized a previously described, conditional model of progressive parkinsonism (TIF-IA^DATCreERT2^ mice), which is characterized by a broad spectrum of behavioral and molecular phenotypes associated with PD^11^. This approach is different from many previous studies, focused mainly on neurotoxin-based models not taking into consideration the progressive nature of PD. Based on the hallmarks of neurodegeneration in this model and its well-defined progression, we chose 3 time points for analysis, 7, 10 and 13 weeks after induction of the mutation (TAM + 7, TAM + 10 and TAM + 13, respectively) in adult mice. Selected time points cover the most critical steps in the evoked neurodegenerative process. As described by Rieker *et al*., animals at TAM + 7 do not show any behavioral PD-like phenotype or significant cell loss, while mice at TAM + 13 are characterized by profound SN/VTA degeneration and a broad spectrum of parkinsonian phenotype^11^. These observations regarding the previously published phenotypic characteristics of TIF-IA^DATCreERT2^ mice were reflected in our experiments as well. The kinetics of behavioral impairment and the association with cell loss was similar in our mice, as assessed via rotarod analysis and TH+ cell loss in VEH-treated TIF-IA^DATCreERT2^ mice compared with their control littermates (Figs 2–3**)**. Additionally, mutant mice at week 13 after tamoxifen injection (TAM + 13) showed visibly impaired behavior in their cages, including tremor and rigidity, as described previously^11^.

To our surprise, the beneficial effects of noradrenergic system stimulation were restricted rather to treatment with REB, not APM. Among the drug-treated TIF-IA^DATCreERT2^ mice, behavioral characteristics were ameliorated by REB, not by APM as reflected by motor performance on the rotarod test at TAM + 13 (Fig. 2A,B). These positive effects of REB treatment were apparently visible on intracellular level, in particular at TAM + 10, a stage where we found less decreased number of SN dopaminergic neurons after REB treatment, and that furthermore corresponds to a beneficial effect of REB on dopamine levels in the striatum (Figs 3, 4A).

Because the availability of transgenic animals is limited, it was not possible to perform experiments with several dosages of each compound. However, the dosages of REB and APM were chosen based on our own previous experience and well-known data from the literature regarding the effects of these drugs^22–27^. In particular, REB has been proven to be highly effective at a dose of 10 mg/kg^24,27^, and APM has been shown to enhance noradrenergic transmission at a dose of 3 mg/kg^23^. However, other reports show that various alpha_2_-AR antagonists can have different dose-dependent effects on noradrenergic stimulated behavior^28^. Notably, in humans at least, APM might have the side effect of reducing multitasking abilities, which might interfere with the ability of the more vulnerable mutant animals to cope with the accelerated rod^21^. Nevertheless, based on the initial behavioral findings not confirming the potential of this drug, we decided to perform further analysis only in the REB-treated animals and their control littermates.

The potential beneficial effects of REB treatment on motor behavior in the TIF-IA^DATCreERT2^ model were corroborated by the analysis of SN/VTA neuronal loss, as visualized by TH+ cell counting (Fig. 3A–D), especially at 10 weeks after induction of the mutation (TAM + 10) in the region of SN. At that time, TH+ cell loss in the VEH-treated TIF-IA^DATCreERT2^ mice was clearly visible, while in the REB-treated mutants, it did not differ much from control littermates. Moreover, there was a significant difference between these two groups (Fig. 3C**)**. At 13 weeks after tamoxifen induction (TAM + 13), both REB-treated and VEH-treated TIF-IA^DATCreERT2^ mice showed clear loss of TH+ cells in the SN. Similar pattern of changes was observed in VTA (Fig. 3D).

Interestingly, a previous work has shown the beneficial effects of REB on non-motor symptoms of PD in the 6-hydroxydopamine (6-OHDA) model^29^. Moreover, L-DOPA did not modify depressive and anxiety-like behaviors in this model^29^. As of yet, we have no evidence of non-motor symptoms in the TIF-IA^DATCreERT2^ mice and this will need to be addressed by future experiments.

It would be rather unreasonable to expect that the enhancement of noradrenaline will stop or even reverse the inevitable changes evoked by an introduced genetic mutation that ablates the transcription factor crucial for RNA polymerase I activity. However, the basis of our model does not diverge from the nature of disease. Loss of TIF-IA causes a higher stability of the transcription factor p53 that plays important role in the response to cellular stress and regulation of apoptosis among multiple other functions^30^. Moreover, loss of TIF-IA results in downregulation of the mechanistic target of rapamycin (mTOR) pathway^11^ and both of these mechanisms were also found to be affected in PD. We have already proven that different cell populations are characterized by different kinetics of cell loss in transgenic models based on TIF-IA ablation^11,31,32^. Moreover, these models have been previously used to test the effects of L-DOPA, p53 and mTOR-dependent signaling pathways on various functions linked to dopamine system integrity, as summarized in the Suppl. Table 1. Based on these evidences, and the results presented here we propose their application to test anti-PD treatments^11^, however it is important to emphasize that we need to consider time-and cell-specific effects. The current study confirms the hypothesis that NA has the potential to be neuroprotective in PD, although the underlying mechanisms need to be further investigated^33,34^.

This idea is also in line with the observation that the drugs targeting noradrenergic and serotonergic systems (i.e., mirtazapine) can be therapeutic against MPTP neurotoxicity in mice, possibly by regulating DA release^35^. To address this issue, we have analyzed *postmortem* levels of DA at two time points – 10 and 13 weeks after tamoxifen injections. We were able to see differentiated effects of REB treatment 10 weeks after induction of the mutation (TAM + 10) as revealed by dopamine content in the striatum of REB-treated and VEH-treated TIF-IA^DATCreERT2^ mice (Fig. 4A**)**. After 13 weeks (TAM + 13) the striatal levels of dopamine were profoundly diminished in all mutants to similar degree despite of drug treatment (Fig. 4A). This is not surprising as the animals at this stage were also shown to have more or less similar extents of TH+ cell loss (Fig. 3A–D), and as already has been mentioned, it would be very unlikely to expect that the effects of the mutation can be postponed for a longer time period. All animals treated with REB were also characterized by higher levels of NA, which might be explained by long term changes evoked by chronic, 21-day treatment with this highly selective NA reuptake inhibitor (Fig. 4D).

Although the beneficial effects shown on motor deficits, neuronal survival and striatal DA content seem to be only temporary, our study points to a differential impact on these by different drugs activating noradrenergic transmission in a novel model of progressive dopaminergic neurodegeneration, proposing its further application for similar studies despite of not a causative mutation. On the other hand, although we have no evidence that loss of TIF-IA causes PD, TIF-IA expression is reduced in PD - there is a number of evidence showing that rRNA synthesis is affected in dopaminergic neurons from PD patients, MPTP-based models, as well as in some genetic-based models of PD^11,36,37^. The mechanism behind that is still poorly understood, nevertheless, these models were shown to be responsive to L-DOPA^11^ and amelioration of the dopaminergic neuronal survival was achieved by the loss of PTEN (crucial phosphatase involved in the regulation of the cell cycle), resulting in the increased activity of the mechanistic target of rapamycin (mTOR) pathway^12^. Nevertheless, here we were able to confirm based on TIF-IA^DATCreERT2^ model that NA is potentially neuroprotective in PD^33,34^. Therefore, this work suggests its application, at least, for testing non-causative therapeutic approaches aiming at improving dopaminergic neuron survival.

There are evidences that VTA and SN cells express also alpha1 adrenoceptors (in particular alpha1A-AR subtype)^38^ which could explain why noradrenaline enhancement can be beneficial in PD. To further explore the mechanistic issue whether the beneficial effects of noradrenergic stimulation might be mediated by alpha1-AR, we have treated primary culture of w/t C57Bl/6 J mouse embryonic midbrain neurons with phenylephrine, a selective alpha1-AR agonist. Obtained data indicate a pro-survival effect in this context (Fig. 5). However, whether this mechanism is directly related to alpha1-AR stimulation remains unsolved as contrary to phenylephrine, REB *per se* was not able to evoke any impact on TH+ cells survival in primary neuronal culture. On the other hand, REB acts mainly as noradrenergic reuptake inhibitor and its receptor activity is only a minor effect^39^. Under these conditions, it seems plausible that only phenylephrine showed pro-survival potential. Of course, since the experiment was performed on primary cultures derived from w/t not mutant mice (the studied model was created using an inducible spatiotemporal mutation, therefore it is not possible to obtain embryos with TIF-IA deletion), we cannot claim that the *in-vitro* experiment fully reflects the situation observed in TIF-IA^DATCreERT2^ mice. Nevertheless, this experiment revealed an additional, important evidence supporting the general concept presented in this paper, that stimulation of noradrenergic transmission can possess beneficial effects in the model of progressive parkinsonism.

As a summary, we can conclude that REB treatment might have beneficial effects in PD. However, it remains unclear whether these effects are simply associated with adaptive changes in response to stimulation of the noradrenergic system or a neuroprotective property of noradrenergic stimulation on dopaminergic neurons. At this stage, we cannot exclude that the improved PD-like phenotype in the REB-treated TIF-IA^DATCreERT2^ mice was, at least partially, associated with adaptive changes after 3-weeks of drug treatment and boosted noradrenergic transmission, which is known to enhance arousal and vigilance and thus might improve the ability to complete the task on rotating wheel. Nevertheless, our results indicate a possible influence of the noradrenergic system on dopaminergic neurons and support the potential of NA as a therapeutic target in PD, which has been suggested by others^10,40,41^.

## Supplementary information

Suppl. Table 1
