# Supplementary material for: Stimulation of noradrenergic transmission by reboxetine is beneficial for a mouse model of progressive parkinsonism
Source: Sci Rep. 2019 Mar 27;9:5262. doi: 10.1038/s41598-019-41756-3 (PMC6437187; doi:10.1038/s41598-019-41756-3)
Supplement: Supplementary file 1 — Suppl. Table 1 [file 41598_2019_41756_MOESM1_ESM.docx]

**Supplementary information**

**Stimulation of noradrenergic transmission by reboxetine is beneficial for a mouse model of progressive parkinsonism.**

Grzegorz Kreiner^1#^, Katarzyna Rafa-Zabłocka^1^, Justyna Barut^1^, Piotr Chmielarz^1^, Marta Kot^2^, Monika Bagińska^1^, Rosanna Parlato^3,4^, Władysława Anna Daniel^2^, Irena Nalepa^1^

^1^ Dept. Brain Biochemistry, Institute of Pharmacology, Polish Academy of Sciences, 31-343 Kraków, Smętna 12, Poland

^2^ Dept. Pharmacokinetics and Drug Metabolism, Institute of Pharmacology, Polish Academy of Sciences, 31-343 Kraków, Smętna 12, Poland.

^3^ Institute of Applied Physiology, University of Ulm, 89081 Ulm, Germany

^4^ Institute of Anatomy and Cell Biology, University of Heidelberg, 69120 Heidelberg, Germany

^#^Correspondence:

Dr. Grzegorz Kreiner

E-mail: kreiner@if-pan.krakow.pl

**Suppl. Table 1: Summary of the approaches used to ameliorate the phenotype of the model based on the conditional loss of TIF-IA in dopaminergic neurons.**

| **Model** | **Phenotypes** | **Pharmacological /genetic approach** | **Read-out** | **References** |
| --- | --- | --- | --- | --- |
| **TIF-IA^DATCre^**  (constitutive) | Conditional loss of TIF-IA in DA neurons during embryonic development, lethal at ca. 90 days | L-DOPA (50 mg/kg, i.p.) injection at 70 days, when less than 20% SN DA neurons are left.  Subcutaneous L-DOPA pellets over 6 weeks | Increased lifespan and weight gain | Rieker et al., J Neurosci **2011**, 31(2):453-60 |
| **TIF-IA^DATCreERT2^**  (inducible) | Inducible conditional loss of TIF-IA in DA neurons in adulthood by Tamoxifen injection | Injection of pifithrin (p53 inhibitor, 2.2 mg/kg body weight) for 6 weeks starting one week after TAM; generation of double mutants lacking TIF-IA and p53 | Increased survival of TH+ neurons at TAM+7 weeks | Rieker et al., J Neurosci **2011**, 31(2):453-60 |
|  |  | Generation of double mutants lacking TIF-IA and PTEN to restore mTOR activity | Increased striatal dopamine, amelioration of gait abnormality and rotarod performance at TAM+10 weeks, independently of any effects on survival of SN TH+ neurons at TAM+7 weeks | Domanskyi et al., FASEB J **2011**, 25(9):2898-910 |
|  |  | Stimulation of noradrenergic transmission by reboxetine | Increased SN TH+ neurons and striatal dopamine content at TAM+10 | This work: Fig. 2, 3 and 5 |
